# Brain-Computer Interface application: auditory serial interface to control a two-class motor-imagery-based wheelchair

**DOI:** 10.1186/s12984-017-0261-y

**Published:** 2017-05-30

**Authors:** Ricardo Ron-Angevin, Francisco Velasco-Álvarez, Álvaro Fernández-Rodríguez, Antonio Díaz-Estrella, María José Blanca-Mena, Francisco Javier Vizcaíno-Martín

**Affiliations:** 1 Department of Electronic Technology, University of Málaga, 29071 Málaga, Spain; 2 Department of Psychobiology and Methodology of Behavioral Sciences, University of Málaga, 29071 Málaga, Spain

**Keywords:** Brain–Computer Interface (BCI), Brain-Controlled Wheelchair (BCW), Two-class, Motor imagery (MI) task, Serial, Auditory interface

## Abstract

**Background:**

Certain diseases affect brain areas that control the movements of the patients’ body, thereby limiting their autonomy and communication capacity. Research in the field of Brain-Computer Interfaces aims to provide patients with an alternative communication channel not based on muscular activity, but on the processing of brain signals. Through these systems, subjects can control external devices such as spellers to communicate, robotic prostheses to restore limb movements, or domotic systems. The present work focus on the non-muscular control of a robotic wheelchair.

**Method:**

A proposal to control a wheelchair through a Brain–Computer Interface based on the discrimination of only two mental tasks is presented in this study. The wheelchair displacement is performed with discrete movements. The control signals used are sensorimotor rhythms modulated through a right-hand motor imagery task or mental idle state. The peculiarity of the control system is that it is based on a serial auditory interface that provides the user with four navigation commands. The use of two mental tasks to select commands may facilitate control and reduce error rates compared to other endogenous control systems for wheelchairs.

**Results:**

Seventeen subjects initially participated in the study; nine of them completed the three sessions of the proposed protocol. After the first calibration session, seven subjects were discarded due to a low control of their electroencephalographic signals; nine out of ten subjects controlled a virtual wheelchair during the second session; these same nine subjects achieved a medium accuracy level above 0.83 on the real wheelchair control session.

**Conclusion:**

The results suggest that more extensive training with the proposed control system can be an effective and safe option that will allow the displacement of a wheelchair in a controlled environment for potential users suffering from some types of motor neuron diseases.

## Background

There are certain diseases that could prevent the patient’s communication with his or her surroundings to the point of not even having control over such seemingly simple actions as precise control of the eyes or breathing (e.g. advanced states of locked-in syndrome [1]). However, researchers have developed systems that are able to establish an additional communication channel between the brain activity of the patient and a particular device. These systems are named Brain–Computer Interfaces (BCIs) [2]. Such devices can be used for many different applications such as writing through a speller matrix [3] or even controlling a robotic arm [4], domotic system [5], or telepresence robot [6]. A wheelchair can be another example of the application of a BCI system, as Millán et al. explained in their review about combining assistive technologies and BCIs [7]. The development of a brain-controlled wheelchair (BCW) that can be handled by such patients would grant them autonomy to move through a controlled environment.

The interest in controlling a mobile device through a BCI system has been growing since the publication of Millán et al., wherein the control of a mobile robot was presented [8]. Tanaka et al. presented the first BCW afterwards [9]. Despite this interest and due to the complexity of developing a BCW, it should be mentioned that the number of scientific contributions in the field is not very high. All these contributions use electroencephalographic (EEG) signals to control the system but differ from each other depending on the type of navigation, the specific signal used for handling the BCI system, the type of participant, the way in which the interface is presented, the tasks to be performed by users, or the number of commands available on the device.

Although there are some BCWs that are controlled by potential P300 (e.g. [10, 11]) or SSVEP (e.g. [12, 13]), most systems make use of endogenous signals and especially sensorimotor rhythm (SMR), which are usually based on the discrimination of different mental tasks [14]. While endogenous signals may require more extensive training to acquire a precise modulation [15] compared to exogenous signals (P300, SSVEP), they have the great advantage of not requiring external visual stimulation [16]. This advantage allows the visual channel to remain dedicated to the maintenance of visual attention on the environment, an important factor when controlling a wheelchair.

We can divide the systems most commonly used in BCW navigation to date into two groups: i) those with high-level navigation, in which the user just needs to select the destination and the BCW performs the navigation of the path, choosing the necessary commands (e.g. [10, 17]) and ii) those with low-level navigation, in which the wheelchair moves through simple navigation commands (e.g. move forward, move backward, turn left, and turn right) selected by the subject (e.g. [9, 18]).

Another important feature is the type of movement that the BCW makes. The control can be performed through continuous movements (e.g. [19, 20]) or discrete ones (e.g.[9, 11, 21]). In the first case, the user controls the extension of the movements. In the second case, the movements are predefined in specific extensions that usually correspond to 90° turns and advances of a certain fixed distance.

To summarize, the research carried out in relation to the control of a wheelchair through brain activity is limited. From existing systems, those based on the discrimination of mental tasks offer more autonomy to subjects. All the studies related to those systems are included in Table 1. As we can see, these systems often associate a navigation command with a specific mental task, resulting in systems that offer few navigation commands or that require a high number of mental tasks to increase the number of commands. However, as suggested by several studies, increasing the number of mental tasks to be discriminated worsens the performance of a BCI system, as it is necessary to discriminate between only two mental tasks to minimize misclassification [22, 23], with the consequent problem that the number of navigation commands is reduced.

**Table 1:** Endogenous brain-controlled wheelchair

Paper	Control	Commands	User’s task	Participants
Tanaka et al. (2005) [9]	Discrete	2	2	6
Millán et al. (2009) [19]	Continuous	3	3	3
Hema, Paulraj, Yaacob, Adom, & Nagarajan (2011) [35]	Mixed^a^	4	4	4
Tsui et al. (2011) [21]	Discrete	3	3	2
Carra & Balbinot (2013) [37]	Discrete	2	2	1
J. Li & Liang (2013) [36]	Continuous	3	3	1
Carlson & Millán (2013) [38]	Continuous	2	2	4

^a^Discrete turns and continuous advance and returns

In order to provide several navigation commands without worsening the performance of the BCI system, the BCI group of the University of Málaga (UMA-BCI) has been working on a paradigm based on the discrimination of only two mental tasks (a mental activation task versus another mental task), allowing the selection of four different navigation commands. This paradigm uses a serial auditory interface, so that subjects hear the available commands and they use one task to select the command, or the other task to let it go. The paradigm has been validated to navigate in virtual environments (VEs) [24, 25] and to control a real robot with continuous movement by providing four different navigation commands: move forward (F), move backward (B), turn right (R), and turn left (L), as well as providing the option of stopping [26].

The main objective of this work is to study whether the proposed paradigm is an effective option to control a BCW in all directions (very preliminary results have already been suggested [27]). The control of the wheelchair will be carried out through the discrimination of the minimum number of mental tasks (i.e., two), helping the learning process and the interaction control. Both of these features are very important if we want to ensure the usability of the system and user safety. To achieve this objective, the subjects will undergo only three training sessions. In this first phase of the study, the movements of the wheelchair will be discrete.

## Method

### Participants and data acquisition

The study initially involved 17 participants (aged 22.12 ± 3.44, 13 males and four females), identified as A1–A12 and B13–B17 here. With the exception of subject A1, none of them had previous experience of BCI systems. Subjects B13 to B17 participated in the preliminary study in [27]. Subjects were recruited through the use of social networks and posters around the campus. The study was approved by the Ethics Committee of the University of Malaga and met the ethical standards of the Helsinki Declaration. According to self-reports, none of the participants had any history of neurological or psychiatric illness or were taking any medication regularly. Participants received monetary remuneration ranging between 5 and 20 € according to which part of the study they successfully completed and all participants provided written informed consent before entering the study. All these subjects participated in an initial calibration session. In this session, a first test regarding the ability of subjects to control two mental tasks was performed. As a design criterion, a conventional limit of 30% in the classification error rate was considered to be the maximum that could allow efficient control of the paradigm; the same limit was used in [28] for efficient communication using a two-class BCI for spelling. In a similar way, this study needed users to have acceptable control of their SMRs, which would enable them to control the BCW. Ten subjects were able to fulfill this criterion and continued with the experiment. As mentioned, an error rate higher than 30% made the control of the BCW very difficult, leading to a sensation of random behavior. Even when users could have improved their performance through training, the aim of our study was not to train subjects to control their SMR, but to test the use of the BCW with users already showing control of their EEG.

The EEG was recorded at a sample rate of 200Hz from two large Laplacian channels (for details see [29]) placed over the right and left sensorimotor areas using the electrode positions: C3, F3, P3, T7 and Cz for one Laplacian channel; and C4, F4, P4, T8 and Cz for the other, according to the 10/20 international system. The ground and reference electrodes were placed at positions AFz and Fz, respectively. Signals were amplified by an actiCHamp amplifier (Brain Products GmbH, Munich, Germany). Neither online nor offline artifact detection techniques were employed.

### Signal processing

As mentioned above, subjects participated in an initial training session (see Calibration Session) for calibration purposes. In this session, we recorded the EEG of each subject performing two mental tasks (80 trials for each task). With this data, an automatic process calculated, for each subject, a reactive frequency band and a classification error rate (detailed below). After processing it, we selected those subjects with classification error rates under 30% and obtained the subject-dependent parameters for the next sessions with feedback. Data processing and feedback generation were based on the procedure detailed in [30]:

- a) The reactive frequency band of each participant was automatically selected from all possible frequency intervals between 5 and 17 Hz (with a minimum bandwidth of 2 Hz). The search for the optimal frequency band was limited to the μ band. Although in some cases it is possible to find subjects whose reactive band belongs to the β band, for simplicity we finally selected the 5–17 Hz band. For each tested frequency interval, feature extraction and classification were carried out, giving a frequency band-dependant error rate as a result. The band that led to the lowest classification error rate was regarded as the subject’s reactive frequency band.
- b) The feature extraction consisted of estimating the average power of the signal from the two EEG channels in the specific frequency interval for each trial. This average was calculated by i) digitally band-pass filtering the EEG using a fifth-order Butterworth filter, ii) squaring each sample, and iii) averaging over several consecutive past samples. A total of 100 samples were averaged, giving an estimation of the band power for several intervals of 500 ms.
- c) The features from both channels for all trials were used to compute the error rate time course of a linear discriminant analysis (LDA) classifier [31] by means of a ten-times ten-fold cross-validation scheme. In this way we obtained the estimated minimum error rate of the classifier for power features from the given frequency band.
- d) Feedback generation: the previously selected frequency band and the obtained features were used to set up the LDA whose classification results determined the feedback ‘L’, which was used in the next sessions. This feedback was computed online every 31.25 ms. All data processing was carried out in MATLAB.

### Navigation application

**Fig. 1:**
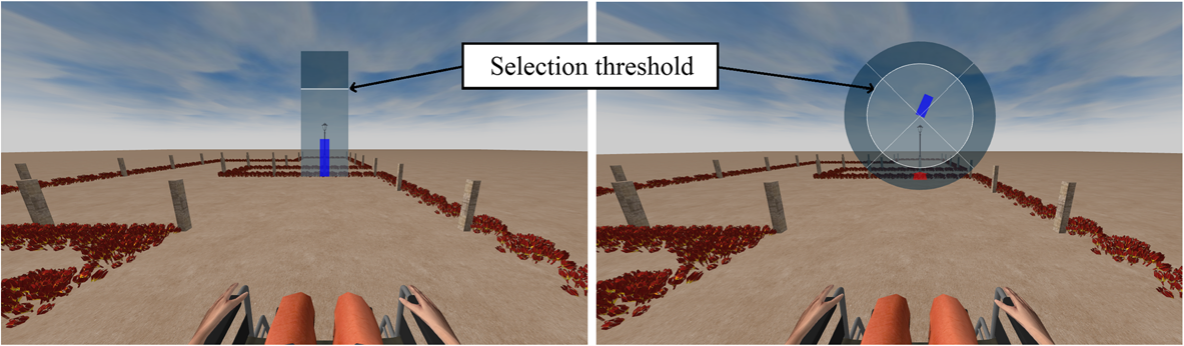
Virtual environment for the first navigation session. NC interface (*left*) and IC interface (*right*)

As mentioned in the Introduction, the navigation paradigm was similar to the one used in our group’s previous publications [24–26]. In this study, one experimental session was carried out in a VE (control of a virtual wheelchair) and another in a real environment (control of a real wheelchair). The same paradigm was used for both virtual and real experimental sessions. In order to provide subjects with an asynchronous (or self-paced) system, the paradigm must produce outputs in response to intentional control as well as support periods of no control [32]; these are the so-called intentional control (IC) and non-control (NC) states, respectively. Both states are supported in the study presented in this paper: the system waits in an NC state in which an NC interface is shown (Fig. 1-left), which enables the subject to remain in the NC state (not generating any command) until he or she decides to change to the IC state, where the control is achieved through the IC interface (Fig. 1-right). The NC interface consists of a semi-transparent vertical blue bar placed in the center of the screen. The bar length is computed every 31.25 ms as a result of the LDA classification: if the classifier determines that the mental task is right-hand MI, the bar extends; otherwise (the other class is idle or rest state) the bar length remains at its minimum size. In order to change from the NC to the IC state, the user must perform the MI task, and so extend the bar, for a certain subject-dependent “selection time” (around 1 s); during this time, the extension of the bar must be kept above a predefined “selection threshold” (70% of the maximum extension). If the length is temporarily (less than a “reset time”) lower than the selection threshold, the accumulated selection time is not reset, but otherwise it is set to zero. This timing is similar for the IC state, but whereas in the NC state the consequence of a selection is a change of state, in the IC the consequence is a navigation action. The IC interface consists of a circle divided into four parts (named command sector onwards), which correspond to the possible navigation commands (F, R, B, L), with a blue bar placed in the center of the circle, which is continuously rotating clockwise. The rotation speed was configurable, but most users fixed it so the bar took 15 s to complete a turn. The subject can extend the bar when carrying out the MI task to select a command when the bar is pointing at it. Once a command is selected, the BCW (virtual or real depending on the experiment) performs a discrete movement: 1 m forward/backward displacement or 90° right/left turn.

The final aim is to control the BCW without visual cues; for this reason, subjects receive audio cues (in Spanish) that represent the situation of the bar in the interface while they interact with the system. One cue indicates the state change from IC to NC: they hear “waiting”. The reverse change is indicated by “forward”, since the forward command is the first available command in the IC state. As mentioned above, once the subject reaches the IC, the bar starts rotating; every time the bar points to a different command, subjects can hear the corresponding word: “forward”, “right”, “backward”, or “left”. Additionally, we included beep sounds that were played when the bar had completed one third and two thirds of each quadrant, in order to inform the participant of the approximate situation of the selecting bar in the quadrant.

In the VE session, two simultaneous control interfaces were provided: visual and auditory. As mentioned before, the objective was to control a real BCW without using a GUI. So in this way, during the first run, users first trained with both interfaces in order to make the learning process of the proposed paradigm easier. Then, in a second run, only the auditory interface was provided. After finishing both runs in the VE session, subjects participated in a second session to control the real BCW using only the auditory control interface.

### Robotic wheelchair

**Fig. 2:**
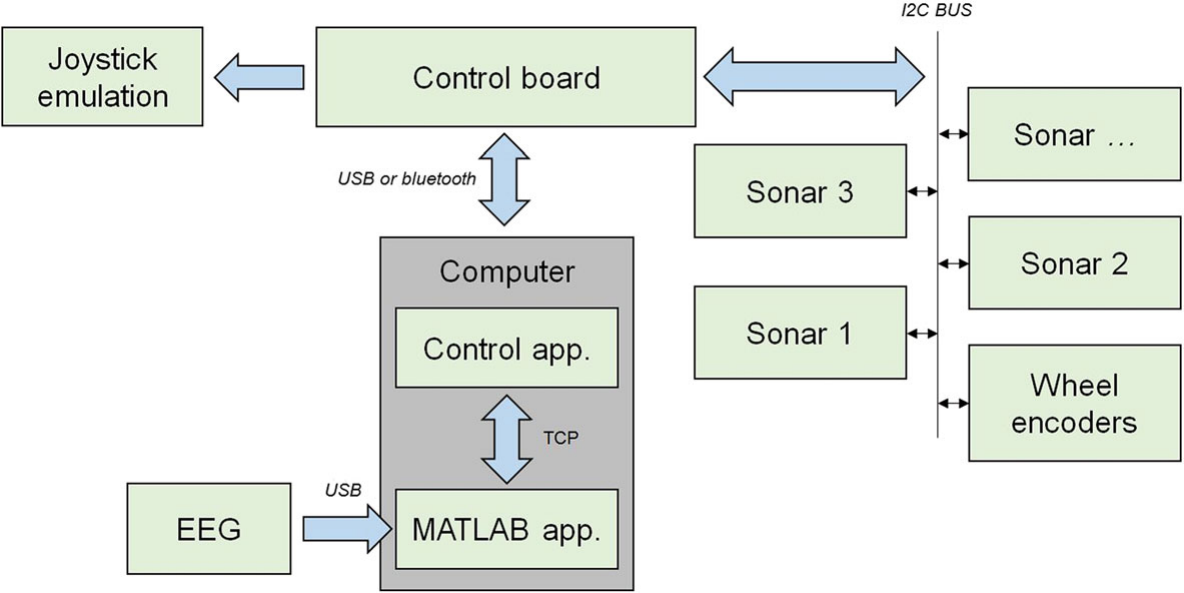
Module structure of the developed brain-controlled wheelchair (further details above in the main text)

The BCW used consisted of a customized Invacare Mistral3 electric wheelchair (see Fig. 2) equipped with a custom-built control board (Control board) that emulated its analog two-axis joystick in real time and received multiple sensor information (Sonars, Wheel encoder) through an I2C bus (I2C BUS). This board was connected through a USB port to a control application (Control app.) written in C that ran on a laptop (Computer). This application received, via a TCP connection, the commands (e.g., move forward) issued by the navigation application running in a MATLAB session, and then transformed them in real time into low-level commands that were fed back to the control board. A set of 11 SRF08 ultrasonic rangefinders (i.e., sonars) allowed the creation of a real-time discrete grid map of the area surrounding the wheelchair, based on the likelihood that a small area near it was occupied. Specifically, when one of the sonars detected an obstacle at a given distance, all the grid cells within its detection cone were updated according to a sonar model, and, in turn, the grid map was also updated in real time. This made it possible to implement different low-level navigation strategies for preventing collisions or for avoiding obstacles by moving along their edges. In this case, only obstacle detection was carried out: When the sensors detected that the center of the BCW was at a distance of 1 m from an obstacle, the system stopped the advance movement. Two AS5048 magnetic rotary encoders were attached to the wheelchair’s driving wheels in order to carry out the odometry and thus compute the wheelchair’s heading at every moment. The application control made use of this information to correct small drifts both online and right after having performed a displacement. As in the case of the virtual BCW, a command selection involved a discrete movement: forward/backward displacement of 1 m or a 90° right/left turn. The BCW took around 10 s to advance 1 m and 15 s to complete a 90° turn (these values could vary slightly because of the mentioned drift corrections).

### Procedure

**Fig. 3:**
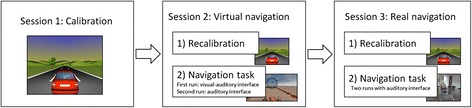
Experimental procedure

The study involved a total of three sessions: i) an initial calibration session, ii) a navigation session in a VE, and finally iii) a navigation session in a real environment with the BCW (see Fig. 3). Both the calibration session and the virtual navigation were carried out in a room where the participant performed the experimental task alone, so that he or she would not be disturbed by external elements, sitting in an armchair in front of a 15.6-inch laptop screen. It should be added that to continue with the next session, subjects had to properly complete the required task in the current session.

#### Session 1: Calibration session

**Fig. 4:**
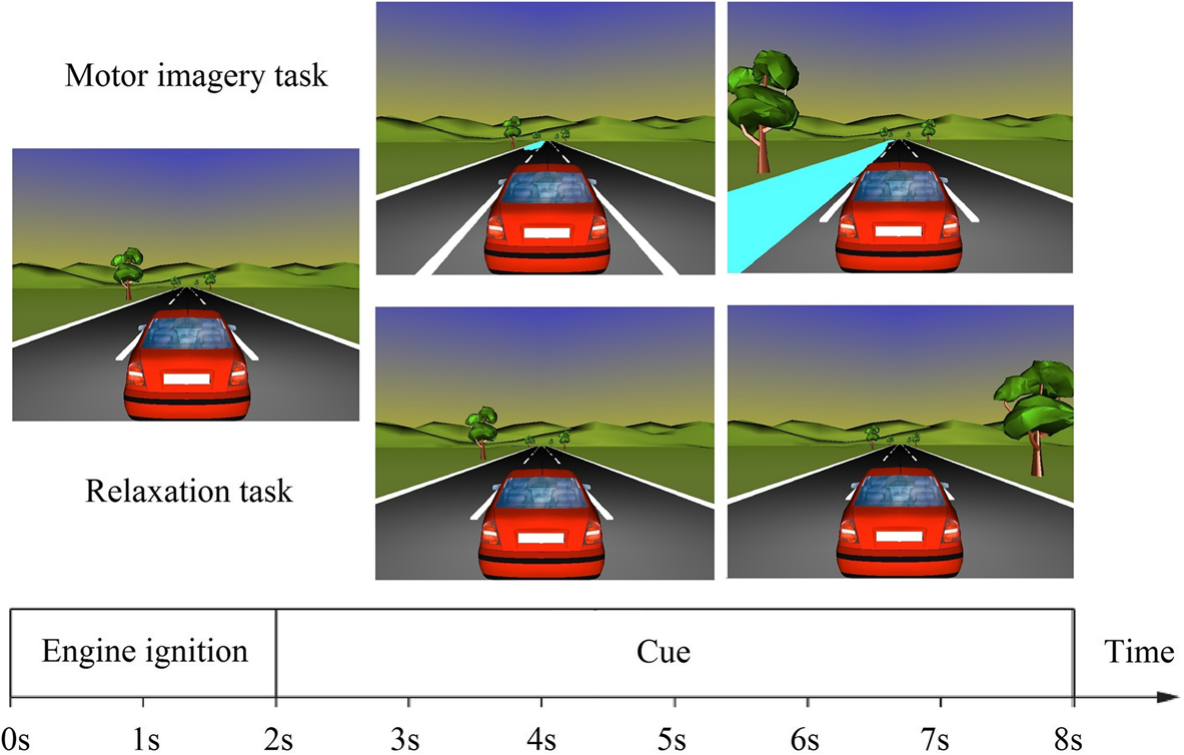
Timing of calibration trials. (*Top*) Right hand MI and (*bottom*) relaxed state tasks

In the first phase, participants were trained to perform the two mental tasks of the BCI paradigm without receiving any feedback. As in [26], in this calibration session subjects had to control the displacement of a virtual car to the right or left, depending on the mental task carried out, in order to avoid an obstacle. Even when there was no feedback at this point, this same scheme was used afterwards in a feedback test prior to the BCW sessions. Each trial was 8 s long and followed the timing shown in Fig. 4. Initially, the car was stopped, the sound of an engine starting indicated the beginning of the trial, and the car started moving forward. Then, at 2 s, the appearance or not of a puddle-like obstacle at the end of the road indicated the mental task to be carried out. If it appeared (always in the left lane), subjects were to imagine right-hand movements. If it did not appear, they were to remain in a relaxed state. At 4.25 s, the puddle was situated beside the car, starting the feedback period in which subjects were able to control the movement of the car to the left or right according to the classification result in order to avoid the obstacle (session with feedback). In sessions without feedback, the car remained in the central lane during the feedback period. The trial finished at 8 s and then started again after a pause ranging from 0.5 to 3 s (randomly distributed). This phase consisted of four blocks of 40 trials each, namely 20 “right-hand MI” trials and 20 “mental relaxation” trials, which were randomly presented. This phase lasted for approximately half an hour, excluding the time needed to set up the EEG recording equipment. Data from this phase were processed by the aforementioned algorithm to obtain the participant’s reactive frequency band and the optimal parameters of the LDA classifier. We excluded those participants whose EEG data at this point could not be classified with an error rate of less than 30%. The virtual car environment was developed with VRML 2.0.

#### Session 2: Virtual navigation

In this session, after preparing the system and explaining to subjects what tasks they should carry out, a short recalibration was performed with only two blocks of data from 40 trials. Differently from the calibration session, subjects received visual feedback on their MI or mental relaxation tasks through the movement of the car. With these data, subjects’ parameters were updated (keeping the same frequency band obtained in the previous session). In this feedback session, the trial paradigm was the same as the one used during the calibration session, except for the fact that now the water puddle occupied part of the middle lane. If the car did not avoid the puddle, a splash sound was heard. In this way we aimed to make this training phase more engaging.

**Fig. 5:**
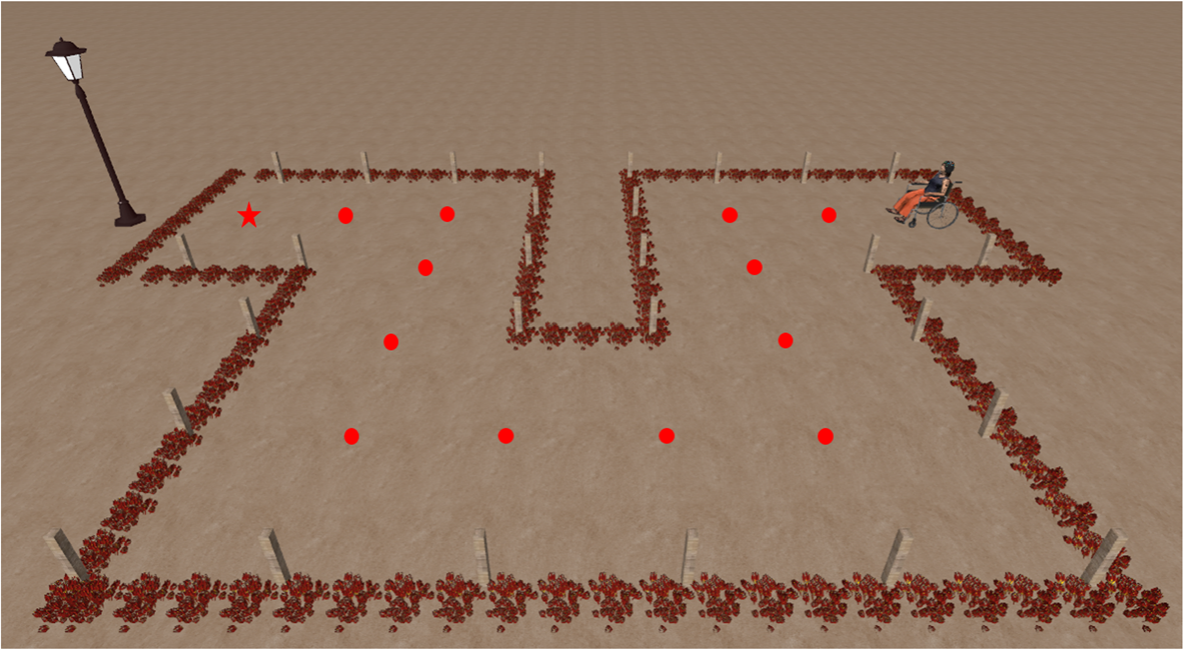
Path to be followed in the virtual environment. Each dot represents one different position where the BCW passes by. Every time the user reaches a new spot, he/she would have to select a new command to continue the path

After the recalibration and before starting the experimental task, subjects trained in controlling the virtual BCW freely for 5 to 10 min in order to adjust some subject-dependent paradigm parameters (such as the bar rotation speed, the selection time, or the ease of bar extension) and thus adapt the interface to each user. As in [26], the experimental trials consisted of two navigation tasks (also called runs) in a VE. Both tasks were the same, with the only difference being that the first had a visual and auditory interface while the second had only an auditory one. The participant saw a plane from a first-person perspective as though he or she were sitting in a virtual wheelchair (see Fig. 1). This wheelchair was at the beginning of a path that was demarcated by flower beds (see Fig. 5). Always using discrete commands (1 m advances and 90° turns), the first task consisted of driving the virtual wheelchair from the starting point to the goal (the end of the path shown in Fig. 5) using the audiovisual interface. Using a red arrow on the ground, the system indicated which command the subject should select. After the selection of a command, the IC interface always presented the bar pointing to the F command. As this training phase was not aimed at assessing user performance, incorrect commands were actually not issued to the virtual wheelchair. In those cases, only an buzzing sound indicating an error was heard and the IC interface was shown, allowing the subject to try again to select the correct command. Completion of the path needed a total of 17 commands: 13 advances, two left turns, and two right turns. This first task with the audiovisual interface was intended to train subjects in the use of the control paradigm. In the second task, they could use only the audio-cued interface, just as would be the case when controlling the real BCW later. The tasks were separated by a short break. At the end of the session, subjects answered a short usability test to evaluate their experiences of using the interface.

#### Session 3: Real navigation

**Fig. 6:**
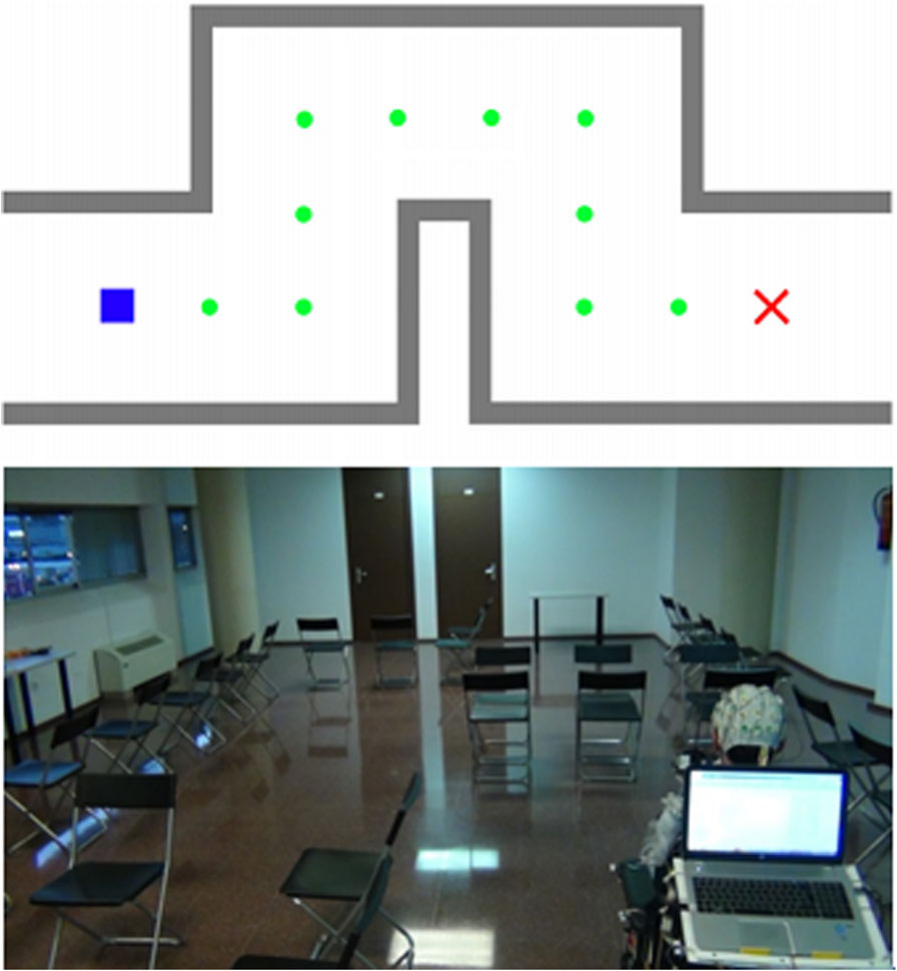
Schematic and real path. (*Top*) The schematic path with the starting point (*blue square*), the different points over which the wheelchair will pass (*green circles*), and the goal (*red cross*). (*Bottom*) The BCW in the real environment. The path to be followed was delimited by folding chairs

Those participants who had completed the training schedule (sessions 1 and 2) attended on a third day to carry out two navigation tasks with the robotic wheelchair. As in the virtual navigation session, a short recalibration was performed before the navigation tasks. This session took place in a private and spacious room in our faculty. Using folding chairs, we demarcated a 2 m wide path that was similar to the one used in the VE, except that this path was 2 m shorter so it could be completed with 11 advances and four turns (see Fig. 6). Its dimensions were adapted to the fixed-magnitude displacements that the wheelchair could perform, that is, 1-m forward/backward movements and 90° turns. If a collision was detected, the control application played the mentioned buzzing sound and if the command issued had been a turn, the system moved the wheelchair back to its previous position. As in the virtual navigation session, subjects could train for around 5 min in controlling the real BCW so that their interface parameters could be adjusted. As in session 2, the experimental trial consisted of two navigation tasks. In the first task, subjects were asked to drive the real wheelchair from the starting point to the goal. Obviously, only the audio-cued interface was used. The second task consisted of returning along the same path to the starting point. As the participant ended the first task by returning to that point, in order to perform the second task it was necessary to make a U-turn at the beginning of it. Differently from the previous virtual session, in this case there were no indications of the proposed commands, and incorrect selections involved a movement of the BCW that users had to amend afterwards. Another difference from the virtual session lay in the fact that after the selection of a command, the bar did not restart from the first command but continued its rotation from the same point at which it had stopped when the command was selected.

### Evaluation metrics and usability tests

Referring to the performance metrics in navigation, in order to accurately assess the performance of users in the management of the BCW, confusion matrix metrics are used (see Eqs. 1–5) as proposed in Mason et al. for self-paced BCIs [33]. In our interface, these parameters are related to when the bar passes through a command sector: it will be categorized as one of the possibilities of the confusion matrix (i.e. true selection, false selection, true non-selection, or false non-selection), depending on the user's intent and what actually happens. The following metrics are used:

i) Recall (see Eq. 1) indicates the user’s ability to select the desired command.
ii) Specificity (see Eq. 2) indicates the user’s ability to avoid unwanted commands.
iii) Precision (see Eq. 3) indicates which of the user’s selections are correct.
iv) Negative Predictive Value (NPV) (see Eq. 4) indicates which of the users’ non-selections are correct.
v) Accuracy (see Eq. 5) shows the level of overall performance.

1 $$ Recall=\frac{{\displaystyle \sum } True\  selection}{{\displaystyle \sum } Selection\  desired} $$

2 $$ Specificity = \frac{{\displaystyle \sum } True\  non - selection}{{\displaystyle \sum } Non- selection\  desired} $$

3 $$ Precision=\frac{{\displaystyle \sum } True\  selection}{{\displaystyle \sum } Actual\  selection} $$

4 $$ Negative\  Predictive\  Value = \frac{{\displaystyle \sum}\mathrm{True}\ \mathrm{non}-\mathrm{selection}}{{\displaystyle \sum } Actual\  non- selection} $$

5 $$ Accuracy=\frac{{\displaystyle \sum } True\  selection+{\displaystyle \sum } True\  non- selection}{{\displaystyle \sum } All\  conditions} $$

**Fig. 7:**
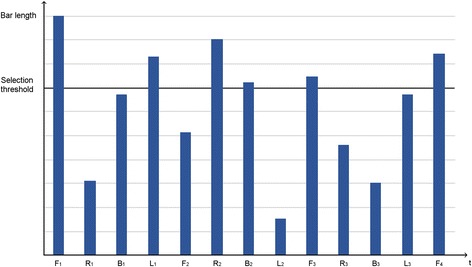
Evaluation example using the confusion matrix related metrics (further details above in the main text)

An illustrative example of a sequence of selections is shown in Fig. 7. The *x*-axis indicates the time elapsed, which in our interface means the rotation of the bar passing through each of the command sectors (F, R, B, L). The *y*-axis indicates the maximum length of the bar during its passage through the corresponding command sector. In this figure, a bar length over the selection threshold indicates a command selection. In this example, the user wants to follow a straight path, which would need two F commands. The first forward command is F1 and the second is F4. Between these two commands, other commands are selected. In fact, this user made two mistakes (L1 and B2) and then corrected them (R2 and F3 for L1 and B2 respectively). In this sequence of commands, the number of true selections was four (F1, R2, F3, and F4), while we found two false selections (L1 and B2), zero false non-selections, and seven true non-selections. This evaluation would lead to the following results: i) Recall equal to 1 (4/4) since the user was able to select all commands that he or she wanted; ii) Specificity equal to .78 (7/9), as out of nine commands that the user did not want to select, he or she did not select seven; iii) Precision equal to .67 (4/6), as four of the six selected commands were desired; and iv) NPV equal to 1 (7/7), since all seven non-selections were desired.

In the virtual navigation task, where the first available command is always Forward command, the percentage of failure for each command and user has been calculated as follow (Eq.6):

6 $$ Failure\left(\%\right) = \frac{F_x}{R_x}\times 100 $$

where ${F}_x$ is the number of failures for a specific *x* command, and ${R}_x$ the total number of times that the mentioned *x* command was required.

Regarding the usability tests, two ad hoc usability tests were designed to evaluate the user experience after navigation sessions (virtual and real). Subjects were asked to evaluate, on a Likert scale of 1 to 5, the following different factors: i) how useful they found the control interface; ii) whether they managed to remain relaxed; iii) whether they felt frustration during the navigation; and iv) how tired they felt at the end of the session. Furthermore, they evaluated the overall interface and the use of the beep sounds. These latter metric was evaluated from 1 in the case of being very detrimental to the performance up to 5 in the case of being very useful. It should be noted that only participants A1–A12 took part in these tests.

## Results

### Calibration session

The most reactive band power features and the minimum error rate obtained for each subject are presented in Table 2. On average, the minimum error rate was 25.70% (±8.87). Of the seventeen subjects, seven (A4, A5, A7, A10, A11, B16, and B17) had error rates above the cutoff point of 30% and were removed.

**Table 2:** Results of the calibration session

Participant	Frequency band (Hz)	Minimun error (%)
A1	9–15	8.44
A2	9–14	18.63
A3	5–17	26.06
A4	11–17	34.00
A5	7–13	35.31
A6	10–15	21.19
A7	7–9	33.88
A8	5–12	12.50
A9	6–13	18.44
A10	9–14	34.63
A11	5–17	30.19
A12	7–12	24.13
B13	6–13	23.00
B14	11–14	20.19
B15	10–13	21.94
B16	10–17	34.31
B17	8–15	40.08
Average	7.94 ± 2.11 to 14.12 ± 2.18	25.70 ± 8.87

### Virtual navigation

In Table 3, the values of different parameters obtained in the virtual navigation session for each subject are shown. The analyzed parameters are: the absolute and relative time taken to complete the path, the total number of fails to select the desired command and divided for each type (F, R, L), the number of times that the NC interface was activated (IC changes), and the different measures obtained for each parameter of the confusion matrix (Recall, Specificity, Precision, NPV, and Accuracy). Participant A3 could not complete any of the tasks, and participant B14 could not complete the second task. Furthermore, thanks to equation 6 referring to the difficulty of the users to select each command, the following average values for failure command (Failure (%)) have been obtained: F: 6.58 ± 4.37; R: 20.32 ± 20.38; L: 37.97 ± 29.82. According to these values repeated measures analysis of variance shown significant differences between commands (F (2, 16) = 6.538; *p* = .008), where the L command was the most difficult for the users to select.

**Table 3:** Results of the users’ performance in the virtual navigation session

Participant	Time lapse		Failed commands				IC changes	Classification matrix’s measures				
	Absolute	Relative	F	R	L	Total		Recall	Specificity	Precision	NPV	Accuracy
	Virtual navigation task 1: visual-auditory interface											
A1	215	1.09	0	0	0	0	0	.94	1.00	1.00	.92	.97
A2	381	1.92	2	0	11	13	0	.85	.67	.57	.90	.73
A6	432	2.18	1	0	0	1	1	.81	.96	.94	.85	.89
A8	312	1.80	1	0	7	8	0	.89	.65	.68	.88	.76
A9	294	1.48	0	1	1	2	0	.94	.86	.89	.92	.91
A12	284	1.43	1	0	2	3	0	.94	.75	.85	.90	.87
B13	413	1.78	1	0	2	3	1	.63	.92	.85	.78	.80
B14	392	1.98	2	1	0	3	0	.81	.80	.85	.75	.81
B15	284	1.43	0	0	4	4	0	1.00	.67	.81	1.00	.86
Average	334 ± 73	1.68 ± 0.34	0.89 ± 0.78	0.22 ± 0.44	3.00 ± 3.78	4.11 ± 4.01	0.22 ± 0.44	.87 ± .11	.81 ± .13	.83 ± 13	.88 ± .08	.84 ± .08
	Virtual navigation task 2: auditory interface											
A1	246	1.24	2	0	0	2	0	.89	.86	.89	.86	.88
A2	513	2.59	0	6	25	31	0	1.00	.39	.35	1.00	.54
A6	410	2.09	1	3	3	7	0	.89	.63	.71	.86	.76
A8	251	1.27	0	1	1	2	0	1.00	.80	.89	1.00	.93
A9	303	1.53	2	0	0	2	0	.85	.85	.89	.79	.85
A12	278	1.40	0	1	0	1	0	.94	.92	.94	.92	.93
B13	644	3.25	4	0	0	4	8	.41	.95	.81	.76	.76
B15	252	1.27	0	0	1	1	0	1.00	.89	.94	1.00	.96
Average	362 ± 148	1.83 ± 0.75	1.13 ± 1.48	1.38 ± 2.13	3.75 ± 8.65	6.25 ± 10.20	1 ± 2.83	.75 ± .36	.79 ± .19	.80 ± .20	.90 ± .10	.83 ± .14

The Relative time is calculated by comparing the Absolute Time and the time obtained on a reference path in which, once a selection is made, the NC interface is presented for the selection of a new command (in this way, a subject can only make one selection per IC interface)

A bivariate correlation analysis through Pearson’s coefficient makes it possible to determine which parameter of the confusion matrix is related to the time required (Relative time) to complete the path. Significant correlations were obtained for the parameters Recall (*r* = −.727; *p* = .026) and Accuracy (*r* = −.81; *p* = .008) but not for the other parameters, that is, Precision (*r* = −.561; *p* = .116), NPV (*r* = −.513; *p* = .158), and Specificity (*r* = −.237; *p* = .539).

### Real navigation

All the obtained parameters in the real navigation session are shown in Table 4. This table is similar to Table 3, except for the command parameters. Table 3 shows the number of fails to select the desired commands for each type, whereas Table 4 shows the total number of times that a specific command was selected, and how many of these commands were correctly selected by the user (Correct) or incorrectly selected (Incorrect). In this session, all the participants completed the first task. Because the duration of the session was limited, subjects A8 and A9 did not participate in the second task, mainly due to the excessive time they took to complete the first task (1111 and 1094 s respectively).

**Table 4:** Results of the users’ performances in the real navigation session

Participant	Time lapse		Selected commands							Classification matrix’s measures				
	Absolute (s)	Relative	F	B	R	L	Correct	Incorrect	IC changes	Recall	Specificity	Precision	NPV	Accuracy
	Real navigation task 1													
A1	236	0.60	11	0	2	2	15	0	0	1.00	1.00	1.00	1.00	1.00
A2	694	1.77	11	1	5	5	16	6	2	.64	.88	.73	.84	.81
A6	674	1.72	12	1	11	4	20	8	0	.87	.68	.71	.85	.77
A8	1111	2.84	12	2	4	4	18	4	5	.55	.95	.82	.83	.82
A9	1094	2.80	17	2	13	10	27	15	1	.77	.69	.64	.80	.72
A12	568	1.45	12	0	7	6	19	6	0	.83	.79	.76	.85	.80
B13	346	0.75	13	0	3	2	17	1	0	.94	.95	.94	.95	.95
B14	489	1.06	14	0	2	2	17	1	0	.89	.95	.94	.90	.92
B15	895	1.94	16	3	8	6	24	9	3	.75	.81	.73	.83	.78
Average	679 ± 308	1.66 ± 0.8	12.5 ± 2.26	1 ± 0.89	7 ± 4.24	5.17 ± 2.71	19.17 ± 4.26	6.5 ± 4.97	1.33 ± 1.97	.80 ± .14	.86 ± .12	.81 ± .13	.87 ± .07	.84 ± .09
	Real navigation task 2													
A1	422	0.90	12	0	6	9	21	6	0	.95	.67	.78	.92	.83
A2	922	1.96	10	3	9	7	16	13	2	.62	.81	.55	.85	.76
A6	677	1.44	12	3	10	8	25	8	0	.93	.68	.76	.89	.81
A12	942	2.00	16	1	12	6	26	9	2	.79	.80	.74	.84	.80
B13	438	0.81	13	1	5	2	19	2	0	.95	.93	.90	.96	.94
B14	500	0.93	14	0	3	5	20	2	0	.95	.89	.91	.94	.93
Average	650 ± 236	1.34 ± 0.54	12.5 ± 2.52	1.75 ± 1.5	9.25 ± 2.5	7.5 ± 1.29	22 ± 4.55	9 ± 2.94	1 ± 1.15	.87 ± .14	.80 ± .11	.77 ± .13	.90 ± .05	.85 ± .07

Subjects B13, B14, and B15 followed a different path from that described. In fact, this path was the same as that used in the virtual navigation session, requiring two more F commands. Therefore, these results were not taken into account when calculating the averages of the non-relative variables such as the Absolute Time lapse and the selected commands

As in the virtual navigation session, we proceeded to analyze the relationship between the Relative time and the different metrics obtained from the confusion matrix. In this case, significant correlations were obtained for Recall (*r* = −.829; *p* = .006), Precision (*r* = −.685; *p* = .042), NPV (*r* = −.926; *p* < .001), and Accuracy (*r* = −.826; *p* = .006) but not Specificity (*r* = −.278; *p* = .469).

### Usability tests

**Fig. 8:**
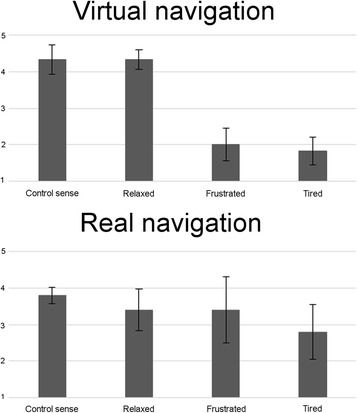
Results in the usability tests. Average values obtained for each answer related to the subjective questionnaire in the virtual (*top*) and real (*bottom*) navigation sessions

The different mean values obtained in the usability tests are shown in Fig. 8. Besides, mean scores of 4.57 ± 0.53 and 4.5 ± 0.55 in the overall assessment of the interface were obtained for virtual and real sessions respectively. Regarding the utility of beep sounds presented at the command interval, the mean scores were 4.57 ± 0.79 and 4.4 ± 0.89 for the virtual and real sessions respectively. Finally, subjects were also asked for the need to display the bar extension in the virtual session, and the mean value obtained was 3.57 ± 0.79.

An interesting analysis is to determine a possible relationship between these usability metrics and the different parameters of the confusion matrix. To this end, a correlation analysis through the Spearman correlation coefficient was performed. In the virtual navigation session, a significant correlation between the sense of control and different parameters of the confusion matrix was obtained: Specificity (ρ = −.878; *p* = .021), Precision (ρ = −.878; *p* = .021), and Accuracy (ρ = −.878; *p* = .021). Regarding the real navigation session, a correlation was found only between the feeling of tiredness (tired) and the Recall parameter (ρ = −.82; p = .046). It is noteworthy that these tests were only carried out by participants who successfully completed at least one task of the corresponding navigation session, that is, those shown in Tables 3 and 4.

## Discussion

Due to the specific paradigms presented by the BCWs shown in Table 1, it is difficult to make an accurate comparison between them and the results obtained in the present work. In addition, these studies should have the same participant experience level, tasks and metrics to allow a direct comparison between them as it has been manifested in previous reviews [14, 34].

In spite of this difficulty to compare the obtained results with those obtained in previous works, it is important to mention some differences. From those studies based on the discrimination of mental tasks for controlling a wheelchair, only one provides 4 navigation commands [35] (move forward, turn left, turn right and stop) however, each command is associated to a specific motor imagery task, being necessary to discriminate between four different mental tasks. In [19, 21, 36] three mental tasks are discriminated in order to execute three different commands (move forward, turn left and turn right [19, 36] or turn left, turn right and stop [21]). Finally, in [9, 37, 38], the discrimination between two mental tasks provide only two different commands (turn left and turn right [9, 38] or turn right and move forward [37]). Our proposed BCI is the only one which offers the command move backward in addition to the commands move forward, turn left, turn right and stop.

As we can check in Table 1, the number of participants for controlling the different BCWs is very reduced (6 [9], 4 [35, 38], 3 [19], 2 [21] and 1 [36, 37]), being the vast majority of them participants with previous experience in BCI (only one participant had no previous experience in BCI [19], and no information is provided regarding the subjects’ experience in [9]). In our study, 17 subjects were initially involved and only one had previous experience in BCI. Out of the 17 users who participated, 10 passed the first training session, obtaining a classification error under the established criterion of 30%. As we can see in Table 2, for these 10 subjects, the minimum error rate ranged from 8.44% (A1) to 26.06% (A3). All these subjects, except for subject A3, were able to control the proposed BCW, being this number of participants much higher than other studies. The fact that subject A3 could not complete any task in the virtual session could suggest that, in order to guarantee the ability to control the BCW, the criterion of achieving a classification error rate of 30% during the calibration session should be reduced to 25%. Once this error rate is obtained, a subject will have high probability of controlling the proposed BCW. Next, the obtained results shown in the previous section will be discussed.

### Virtual navigation

As can be observed in Table 3, the obtained results are very positive, with an average Accuracy of above .80 in both tasks (.84 ± .08 and .83 ± .14 for tasks 1 and 2 respectively). The success in the execution of the path in both tasks shows the effectiveness of the proposed training paradigm: not only did the paradigm seem a good option for helping subjects learn to control the interface but also they seemed to be able to manage the system using only the audio interface.

Regarding the time needed to carry out the task, only some users required a Relative time close to or above 2 to complete the path: A2 (1.92), A6 (2.18), A8 (1.80), B13 (1.78), and B14 (1.98) in task1 and A2 (2.59), A6 (2.09), and B13 (3.25) in task 2. Except for subject B14, subjects with these values of Relative time were those who had to select the interface control more than once (IC > 1) or made many incorrect command selections (Total > 5). In some ways, these results (i.e., the ability to execute the task) are closely related to the different confusion matrix measures.

It might be considered that a high ability in selecting the desired command (i.e., Recall = 1) would be sufficient to assure a good ability to execute the task; however, to achieve good control, the Precision parameter must be high too. In Table 3, those subjects with Recall = 1 are B15 in task 1 and A2, A8, and B15 in task 2. Of these subjects, only the one with low Precision had a high Relative time value (i.e., subject A2 with Precision = .35). Indeed, a low value of Precision is obtained when the system (i.e., the user) sensitivity to select commands is too high (when it is easier to select a command) and therefore the number of unwanted selections is increased (31 for subject A2). On the other hand, a low value of Recall is obtained when a subject has great difficulty in selecting a desired command. In this case, the bar might make a complete turn without any command being selected and causing an IC change. In Table 3, subjects with IC > 1 are those with the lowest Recall values: A6 (Recall = .81, IC = 1) and B13 (Recall = .63, IC = 1) in task 1 and B13 (Recall = .41, IC = 8) in task 2.

While the Recall and Precision parameters are related to the desired command selections, the NPV and Specificity parameters are related to the desired command non-selections. A value of NPV = 1 means that the total number of non-selected commands was equal to the number of desired non-selections. Therefore, a high value of NPV could be considered as a good control; however, to this end, a high value of Specificity is also required. A small value of Specificity, that is, less than .5, indicates that from the total number of commands that should not be selected, less than half were finally not selected and thus more than half produced failed commands (incorrect selections). These small values of Specificity are obtained when a command is easily selected by the system (i.e., when the bar is easily extended). To avoid this, it is very important to correctly adjust all the parameters related to the bar extension. However, this is not always an easy task, being closely related to the control capacity of the subject. In Table 3, those subjects with NPV = 1 were B15 in task 1 and A2, A8, and B15 in task 2. Of these subjects, only A2 obtained a Relative time close to 2 (2.59), which is explained by his or her low value of Specificity (.39). Although this subject did not fail to select any F commands, he or she failed to select 6 R and 25 L commands. It should be noted that in the virtual session the bar always started in the F command position so the user could select it easily. However, taking into account the distribution of commands, to select the L command, three previous commands had to be avoided (F, R, and B), and this task was difficult for subjects with low values of Specificity. As can be observed in Table 3, those subjects with smaller values of Specificity were those with higher rates of failure to select desired commands (total): A2 (Specificity = .67, total = 13), A8 (Specificity = .65, total = 8), and B15 (Specificity = .67, total = 4) in task 1 and A2 (Specificity = .39, total = 31) and A6 (Specificity = .63, total = 7) in task 2. On the contrary, the only subject with Specificity = 1 was the only one who did not fail to select any desired command (A1 in task 1).

These facts about the time taken to complete the path are confirmed by the observed correlations between the Relative time and the metrics of the confusion matrix, where greater negative significant correlations of Accuracy (*r* = −.81; *p* = .008) and Recall (*r* = −.727; *p* = .026) were found. With regard to Accuracy, this was expectable as it is the general metric of the performance classification. However, thanks to the strong relationship between Recall and time, and not between time and other measures used, it seems that for a faster execution of the path, it is suitable that the interface has a good detection of the desired commands, although this could involve some wrong selections.

### Real navigation

It must be mentioned that, as explained in Procedure, the experiments carried out in session 2 (virtual navigation) and session 3 (real navigation) had significant differences, so the results obtained are not comparable. Not only the length of paths but also the interface paradigms were different. For example, in the virtual session, once a command was selected, the bar position was automatically situated at the F command; however, in the real session, it continued its rotation from the same position. Another important difference is the fact that in the virtual session wrong commands did not execute any movement. Anyway, the purpose of the virtual session was always to act as a training session. As can be observed in Table 4, the results show the good performance obtained by the subjects, with the average values of Accuracy being .84 ± .09 and .85 ± .07 for tasks 1 and 2, respectively. In fact, these average values of accuracy was higher than those obtained in other BCW studies which use discrete commands (80% in [9] and 65.7% in [37]). Although all the subjects achieved high performances on Accuracy (between .72 and 1), regarding the times needed to complete the task, the results were more heterogeneous. Indeed, while some users needed less than 4 min to complete the task, others spent more than 15 min, depending on their initial ability to control their mental activity.

In this real session, subjects with Relative time values close to or above 2 were those with IC > 1: A2 (Relative time = 1.77, IC = 2), A8 (Relative time = 2.88, IC = 5), A9 (Relative time = 2.8, IC = 1), and B15 (Relative time = 1.94, IC = 3) in task 1 and A2 (Relative time = 1.96, IC = 2) and A12 (Relative time = 2.0, IC = 2) in task 2. Once again, IC values >1 are closely related to low Recall values (subjects A2, A8, A9, and B15 are those with the lowest Recall values in task 1 while subjects A2 and A12 are those with the lowest Recall values in task 2). Likewise, subjects with the lowest Precision values are those with the highest number of incorrect commands (Incorrect > 6): A6 (Incorrect = 8, Precision = .71), A9 (Incorrect = 15, Precision = .64), and B15 (Incorrect = 9, Precision = .73) in task 1 and A2 (Incorrect = 13, Precision = .55) and A12 (Incorrect = 9, Precision = .74) in task 2. As mentioned in the previous sub-section (Virtual navigation), incorrect selection of commands is also in concordance with the Specificity parameter. It can be observed in Table 4 that the subjects with the lowest number of Incorrect commands Specificity are those with the highest Specificity values: A1 (Incorrect = 0, Specificity = 1), A8 (Incorrect = 4, Specificity = .95), B13 (Incorrect = 1, Specificity = .95), and B14 (Incorrect = 1, Specificity = .95) in task 1 and B13 (Incorrect = 2, Specificity = .95) and B14 (Incorrect = 1, Specificity = .95) in task 2.

In general the obtained results are very promising, with the lowest value of Accuracy being .72 (subject A9 in task 1). Even those subjects who did not participate in the second task due to the excessive time taken to complete task 1 obtained good performance: A8 (Accuracy = .72) and A9 (Accuracy = .80). It should be noted that some subjects achieved a Relative time < 1, showing their high ability to control the wheelchair: A1 (Relative time = 0.6) and B13 (Relative time = 0.75) in task 1 and A1 (Relative time = 0.9), B13 (Relative time = 0.81), and B14 (Relative time = 0.93) in task 2. Of these subjects, subject A1 made a perfect itinerary in task 1 with no incorrect selection and all matrix parameters equal to 1. What is more, an interesting case may be subject A8 in task 1. In spite of having an Accuracy of .82, this subject took the longest time to complete the path (1111s). These results can be understood if we consider his or her Recall and Specificity parameters. On one hand, the low value of Recall obtained (.55) reveals this subject’s difficulty in selecting the desired command, which caused many IC changes (five) and increased the time taken. On the other hand, his or her high value of Specificity (.95) shows his or her ability to avoid selecting undesired commands. In fact, these values correspond to an unbalanced system whose selection parameters were difficult to adjust.

Regarding the correlations observed between the Relative time taken to complete the path and the metrics of the confusion matrix, larger negative correlations was found for NPV (*r* = −.926; *p* < .001) and Recall (*r* = −.829; *p* = .006). These metrics take into account the number of false non-selections, that is, those cases where the user wants to select a command and does not and therefore has to wait for the bar to complete another turn before selecting it, increasing the time taken considerably. On the other hand, it is remarkable to note the low non-significant correlation between the Relative time and Specificity, which, as in the virtual navigation session, could indicate that it would be more efficient to sacrifice part of the Specificity in order to improve the Recall and thus the time taken to complete the path. As a consequence, the user would lose the ability to avoid selecting unwanted commands but could select the desired commands easily, including both the initially desired command and the corrective command if necessary. Despite being more efficient in terms of time, it is noteworthy that this configuration is not the best taking into account the movements made by the BCW or the user experience.

### Usability tests

In the virtual navigation session, most users expressed a good control sense, and low levels of frustration and tiredness (see Fig. 8). In the real navigation session, similar scores were obtained excepted for the parameters “frustrated” and “tired”. For these factors, a larger variability was obtained, with subjects A6 and A9 reporting the maximum frustration scores. These results are in accordance with the fact that the virtual and real navigation tasks were different. Indeed, in the virtual session, wrong commands were not executed by the system and thus subjects did not have to correct them, making it easier to use compared to the real navigation session.

By analyzing the correlations, it can be seen that the users’ Control Sense levels in the virtual navigation session are related to the general performance of the classification through the significant correlation with the Accuracy and the amount of unwanted commands selected, that is, the number of errors made in the selection of incorrect commands, through the significant correlations with the Specificity and Precision. However, in the real navigation tasks, it was possible to observe a significant relation between the feeling of tiredness and the percentage of incorrect command selections, through the negative significant correlation with the Recall.

## Conclusions

A brain-controlled wheelchair has been developed. In order to provide several navigation commands without worsening the performance, a paradigm based on the discrimination of only two mental tasks has been proposed. The obtained results with healthy subjects demonstrate that this BCW allows them to freely control the wheelchair in four directions and to do so efficiently with only three training sessions. Although the times needed to complete the task were heterogeneous, all the subjects achieved high performance in terms of Accuracy (between .72 and 1).

We observed that subjects who finally successfully controlled the wheelchair were those who initially had adequate initial control of their SMRs in the calibration session (minimum error rate < 25%). However, it is mentioned in some studies that the ability to modulate the SMR can be acquired by users through training (e.g. [15, 39]). In this sense, with more training sessions, not only would better BCW performance be achieved, but also the number of subjects who could control the wheelchair would increase.

Compared with other BCWs, the control strategy proposed in this study seems to allow efficient control of the wheelchair without requiring long training or large mental effort. Besides, because the system is achieved by discriminating only two mental tasks, misclassification is minimized, which is very important in order to guarantee the usability of the system and the user’s safety.

Future work will focus on training users with low initial performance over several sessions until they achieve good control of the wheelchair. Another important objective will be to evaluate the performance of the system with continuous movement.

Thanks to the metrics displayed in the confusion matrix about the performance of the user controlling a BCW, new ways of adjusting the user parameters of our control interface (i.e. the facility to extend the bar or the time needed to maintain the bar above the selection threshold) have been identified in order to make the BCW an efficient proposal with dynamic and efficient management.

## Abbreviations

BCI: Brain-computer interface
BCW: Brain-controlled wheelchair
EEG: Electroencephalogram
IC: Intentional control
LDA: Linear discriminant analysis
MI: Motor imagery
NC: Non-control
NPV: Negative Predictive Value
SMR: Sensorimotor rhythm
VE: Virtual environment
